# Factors associated with adherence to guidelines in cancer pain management among adult patients evaluated at oncology unit, in the University of Gondar Comprehensive Specialized Hospital, Northwest Ethiopia

**DOI:** 10.3389/fpain.2022.884253

**Published:** 2022-08-01

**Authors:** Anteneh Ayelign Kibret, Haileab Fekadu Wolde, Meseret Derbew Molla, Hailu Aragie, Dagnew Getnet Adugna, Ephrem Tafesse, Endalkachew Belayneh Melese, Yilkal Belete Worku, Daniel Gashaneh Belay

**Affiliations:** ^1^Department of Human Anatomy, School of Medicine, College of Medicine and Health Sciences, University of Gondar, Gondar, Ethiopia; ^2^Department of Epidemiology and Biostatistics, College of Medicine and Health Sciences, Institute of Public Health, University of Gondar, Gondar, Ethiopia; ^3^Department of Biochemistry, School of Medicine, College of Medicine and Health Sciences, University of Gondar, Gondar, Ethiopia; ^4^Department of Internal Medicine School of Medicine, College of Medicine and Health Sciences, University of Gondar, Gondar, Ethiopia

**Keywords:** cancer pain, Ethiopia, factors, adherence to guidelines, University of Gondar

## Abstract

**Introduction:**

Cancer pain is one of the most important deleterious and distressing symptoms suffered by patients with cancer which disturb their quality of life, especially in the last part of their life. Alleviating pain is a primary goal of prognosis of cancer pain management and pain symptoms must be prevented, treated as a priority, and considered an independent part of cancer management. Despite the presence of guidelines for cancer pain management, many patients with cancer are still undertreated. Therefore, this study aimed to assess factors associated with adherence to guidelines in cancer pain management among adult patients evaluated at the oncology unit, in the University of Gondar Comprehensive Specialized Hospital (UoGCSH), Northwest Ethiopia.

**Methods:**

An institution-based cross-sectional study was conducted from January to March 2021. All patients who were in cancer treatment were our population of interest. A systematic random sampling technique was used to select a total of 384 participants. The dependent variable of the study was adherence to guidelines in cancer pain management. It was determined using the pain management index (PMI) which was calculated by subtracting the pain level from the analgesics level. A negative range was considered an indicator of poor adherence to guidelines in cancer pain management. Bivariable and multivariable binary logistic regression analyses were performed. Adjusted odds ratio (AOR) with a 95% CI was used as a measure of association. Variables having *P* < 0.05 from the multivariable analysis were considered to have a significant association with the outcome.

**Results:**

The prevalence of poor adherence to guidelines in cancer pain management among 384 adult patients in this study was 21.35% (95%CI: 17.53, 25.76). Patients who were not married [AOR = 2.2; 95%CI: 1.15, 4.19], who know their diagnosis before 4 months ago [AOR = 0.53; 95%CI: 0.26, 0.96], who have metastasis cancer [AOR = 3.76; 95%CI: 1.83, 7.72], and being stage III patients [AOR = 3.21; 95%CI: 1.64, 7.93] and stage IV patients [AOR = 1.63; 95%CI: 1.09, 5.81], respectively, had a significant association with poor adherence to guidelines in cancer pain management.

**Conclusion:**

The prevalence of poor adherence to guidelines in cancer pain management among adult patients with cancer in UoGCSH Northwest Ethiopia is relatively low as compared with other studies. Factors such as patients who were not married and who have metastasis cancer, and being patients with stage III and stage IV cancer had a significant positive association with poor adherence to guidelines in cancer pain management, on the other hand, patients who know their diagnosis 4 months ago had a positive association with having adherence to guidelines in cancer pain management. Patients with high stage and metastasis need care from pain specialists early on in the diagnosis of pain. The hospital should reassure the diagnosis of cancer for the patient before they started the treatment.

## Introduction

Cancer is becoming an increasing public health problem, and an estimated 4% of all deaths, in Ethiopia (1). Pain is one of the most feared symptoms of patients with cancer which occurred throughout their clinical course due to either cancer itself or the cancer treatment (2). A recent study reveals that two million people suffer from pain every day worldwide and cancer pain is one of the major neglected public health problems (3). Studies in some countries of Africa including Ethiopia revealed that the prevalence of cancer pain ranges from 35.7 to 91.6% (4–6).

Pain management is an essential part of oncology care to improve the quality of life of patients with cancer since pain is a major source of suffering (7). There are several guidelines for cancer pain management (8). The WHO analgesic ladder (WHO-AL) is the most common guideline developed in 1986 (revised in 1996) and has been used for the last 20 years (8, 9). It was majorly aimed at decreasing the prevalence of inadequate analgesia (8, 9). According to the WHO guidelines, adequate cancer pain management is when the patient's reported level of pain is similar to the potency of the prescribed analgesic drug which is measured and compared by the pain management index (PMI) (9, 10). On the other side, poor adherence to guidelines in cancer pain management is having a negative PMI score which occurs when the potency of the prescribed analgesic is lower than the level of pain (10). With proper use of this WHO-AL, approximately 88% of patients reportedly obtain reasonable pain relief (9). This stepwise approach from bottom to top cancer pain management is preferably used for chronic pain. The strongest analgesic (for that intensity of pain) is the initial therapy for acute pain and is later toned down (11). Moreover, adjuvant and non-pharmacological treatments are recommended for the therapy path for treating persistent pain in combination with analgesics or other medications (11). A better management approach to cancer pain will not only alleviate the pain symptoms but will also increase the patient's quality of life (12).

Despite the presence of guidelines for cancer pain management, many patients with cancer are still under treatment (13–15). The prevalence of undertreated pain among patients with cancer reach 70% in Japan and 77% in Punjab, India (9). In Ethiopia, the prevalence of poor adherence to guidelines in cancer pain management was 43.95% in Ayder Comprehensive Specialized Hospital and 65% in Gondar University Hospital (16).

Different factors contributed to poor adherence to guidelines in cancer pain management, such as the differences in socioeconomic status, inequality of access to a doctor and medication, inappropriate use, and fear of opioids due to patients' cultural attitudes regarding pain and the use of opioid medications (13–15). Studies also showed that factors such as sex (17), presence of metastasis (16, 18), comorbidity (18), stage of cancer (18), educational level (16–18), and monthly income (18) have a significant association with poor adherence to guidelines in cancer pain management. The WHO estimates that 80% of the world population has insufficient access to appropriate opioid analgesics such as morphine (19, 20). For patients with advanced-stage cancer, liberal use of opioids is always suggested, but a conservative approach to opioid use in this stage may result in poor adherence to guidelines in cancer pain management (9).

As far as our search, despite there being only two studies in the country that tried to assess the prevalence of adequate cancer pain management (16, 18), there were small sample sizes (<100) which makes it difficult to generalize for all source populations.

Therefore, this study aimed to assess the prevalence and factors associated with poor adherence to guidelines in cancer pain management among adult patients evaluated at the oncology unit, in the UoGCSH, Northwest Ethiopia with an adequate sample size. Moreover, the association of poor adherence to guidelines in cancer pain management with different types of variables such as sociodemographic, psychosocial, behavioral, and clinical factors was assessed simultaneously. The result of this study may contribute to the hospital and to the country in drawing the attention of the policymakers, healthcare managers, and healthcare professionals to strengthen effective cancer pain management and provide comfort to patients with cancer. We believe this study highlights the importance of cancer pain management and encourages providers to investigate the true status of cancer pain management.

## Methodology

### Study design and period

The institution-based cross-sectional study was conducted from January to March “2021” among patients with cancer who come to the Oncology ward at the University of Gondar Comprehensive Specialized Hospital. The hospital was established in 1954 and it is located in Gondar town, Central Gondar administrative zone, Amhara National Regional State, which is far about 750 km Northwest of Addis Ababa (the capital city of Ethiopia). Currently, Gondar town has one referral hospital and eight government health centers. University of Gondar Comprehensive Specialized Hospital (UoGCSH) is a teaching hospital, which serves more than 10 million people in the Central Gondar zone and people of the neighboring zones. The hospital has Oncology ward since 2014. The ward currently serves around 3,000 patients with cancer per year. The oncology unit of UoGCSH currently has 30 beds for the management of patients with cancer.

### Source and study population

The source population for this study was all adult patients with cancer who come to both outpatient and inpatient oncology departments at UoGCSH. Those adult patients with cancer who visited the treatment centers between 10 January and 10 March 2021, were the study population. However, adult patients with cancer who are unable to communicate and with severe psychiatric problems were excluded from the study.

### Sample size and sampling procedure

#### The sample size for the prevalence objectives

The sample size for this study is determined using a single population proportion formula by considering the prevalence of 43.95% from the study done in the same setting 5 years ago (18), 95% CI, 5% margin of error.


$$\begin{array}{l} n=\frac{{\left(Z_{a/2}\right)}^{2}pq}{d^{2}},n=\frac{{\left(1.96\right)}^{2}\left(0.44\times 0.56\right)}{{\left(0.05\right)}^{2}}=378 \end{array}$$

Adding 5% non-response, the final sample size was 397.

Of the annual 3,000 patients with cancer attending the outpatient department, proportionally 750 patients are expected to attend in 3 months from 10 January to 10 March 2021. Therefore, when dividing the total number of expected patients in 3 months by the total sample size (750 /397 = 1.85), we get the interval *k* = 2. Therefore, using systematic random sampling, we selected our samples for every two patients.

### Study variables

The outcome variable of this study is poor adherence to guidelines in cancer pain management. If the patient has poor adherence to guidelines in cancer pain management based on the operational definition, it was coded as “1” for yes, unless coded as “0” for no (adherence to guidelines in cancer pain management). On the other hand, the independent variables were sociodemographic variables such as age, sex, marital status, residence, and income status were included. Psychosocial and behavioral factors such as social support, anxiety, cigarette smoking, alcohol drinking, chat-related substance, and physical exercise were considered. Clinical factors such as type of cancer, stage of cancer, duration from diagnosis, presence of comorbidity (HIV, DM, and HTN), treatment modalities, type of analgesics, presence of metastasis, and pain grade were included.

### Operational definitions

#### Cancer pain

Cancer pain was assessed using items two to five of the Brief Pain Inventory-Short Form (BPI-SF) (21). Patients were asked to grade their worst, least, and average pain in the last 1 week and the pain they feel currently. The scoring for each item was from 0 to 10. No pain is indicated by 0 and 10 indicates the severest form of the pain which is explained as “Pain as bad as you can imagine.” Then the pain severity score was calculated by adding the scores from the four items and dividing them by four and the final was classified as 0 for no pain, 1–3 for mild pain, 4–7 for moderate pain, and 8–10 for severe pain. Then finally recoded as 0 for no pain, 1 for mild pain, 2 for moderate pain, and 3 for severe pain (22).

#### Analgesics level

The analgesics given to the participants were classified into 4 categories based on their potency: 0 for “no order analgesics,” 1 for “no opioid analgesics” (e.g., NSAID or acetaminophen), 2 for weak opioids (e.g., codeine), and 3 for strong opioids (e.g., morphine) (10).

#### Poor adherence to guidelines in cancer pain management

Poor adherence to guidelines in cancer pain management was determined using PMI. Cancer pain management is said to be poor adherence when there is a negative PMI score which occurs when the potency of the prescribed analgesic is lower than the level of pain (10).

The PMI is a well-validated technique used to assess the adequacy of pain management (9). PMI was calculated by subtracting the pain level from the analgesics level and ranges from −3 to 3. According to the WHO guidelines, good adherence to guidelines of cancer pain management is when the patient-reported level of pain is similar to the potency of the prescribed analgesic drug (10, 23). The negative scores in a negative PMI range were considered an indicator of poor adherence to guidelines in cancer pain management (10, 23).

#### Anxiety

Generalized Anxiety Disorder 7-item (GAD-7) scale was used to measure anxiety (24, 25). The GAD-7 questionnaire is used to assess problems the respondent bothered in the past 2 weeks that used a measure of generalized anxiety disorder. The items measure the frequency of symptoms on a scale from 0 (not at all) to 3 (nearly every day). When adding the scores of all seven items provide the GAD-7 total score ranging from 0 to 21. Then we used cut-points of ≥5 score for having anxiety (25).

#### Social support

Oslo social support scale (OSSS-3) was used to assess social support. OSSS-3 has three items measured by Likert scales, which are summed to 14 points and categorized as “poor” if the total score is 3–8, moderate is 9–11, and strong is 12–14 (26).

#### Physical exercise

Physical activity was assessed according to WHO steps, by which any movement of the body produced by skeletal muscle, which requires energy expenditure, was taken as physical activity. Thus, physical activity was categorized into three levels: vigorous, moderate, and inadequate or poor physical activity. A vigorous-intensity activity was defined as any activity that causes a large increase in breathing or heart rate (e.g., running, carrying, lifting heavy loads, digging, and construction work) that continues for at least 30 min for a minimum of 3 days per week. The moderate-intensity activity was defined as any activity that causes a small increase in breathing or heart rate (brisk walking or carrying light loads) that continues for at least 30 min for at least 3 days per week, or 5 or more days of these activities for at least 20 min per day, or ≥3 days of vigorous-intensity activity per week for at least 20 min per day. Low-level (sedentary) physical activity was defined as an individual having a physical activity that does not meet any of these criteria (27).

### Data collection procedures

Data were collected using pretested and structured interviewer-administered questionnaire and from a chart review. The questionnaire consists of three parts. The first is for sociodemographic and behavioral characteristics. The second part is for questions about the clinical and medical history of a patient. The third part was focused on the questionnaire to assess social support and the final part of the questioner were contained questions to assess anxiety, cancer pain, and the adequacy of the treatment. Information related to cancer pain and adequacy of treatment will be collected using the BPI-SF (21) which consists of 8 items. The first item is to identify where they felt pain and items two to five are to assess the pain severity. Item six and seven are to assess the type of analgesics used and the adequacy of pain management and the last part is to measure the interference of the pain in daily activities. Information on the variables like type and stage of cancer, type of treatment, and type of analgesics were collected from patient charts. The data were collected by nurses who have a BSc degree and working in the oncology ward and they were supervised by the principal investigator.

### Data quality assurance

The quality of data was ensured through training of data collectors and supervisors, close supervision, and prompt feedback. The training consisted of instruction on interview techniques as per the prepared tool. The data were checked for any inconsistencies, coding errors, out of range, completeness, accuracy, clarity, missing values, and appropriate corrections were made by the principal investigator and the supervisor consistently on the daily basis.

### Data processing and analysis

The survey data were entered into EPI-INFO version 7 and analyzed by STATA 14 software. Descriptive statistics are presented using texts, graphs, and tables. A binary logistic regression model was used to identify factors affecting adequate cancer pain management. Both bivariable and multivariable logistic regression models were carried out. Variables with a *P* < 0.2 in the bivariable analysis were entered into the multivariable analysis. Both crude odds ratio (COR) and adjusted odds ratio (AOR) with 95% CIs were estimated to show the strength of associations. Finally, a *P* < 0.05 in the multivariable logistic regression analysis was used for the interpretation of the results. The Hosmer and Lemeshow goodness-of-fit test was used to test the fitness of the model and it was non-significant (*p* = 0.87).

### Ethical considerations

Ethical approval was obtained from the University of Gondar Institutional Ethical Review Board Committee with reference number 0345/2021. A support letter was obtained from the University of Gondar Research and Community Service and the internal medicine department. Participants were informed about the purpose, objectives, and their right to and not to participate in the study. The privacy and confidentiality of the study participant were ensured by not using a personal identifier. Written informed consent was obtained from the study participants.

## Results

### Background characteristics of study subjects

A total sample of 384 patients with cancer was included in this study with a response rate of 96.7% (384/397). Nearly half (51.04%) of study subjects were found in the age group of 18–49 years, with a median age of 48.5 (IQR: 40, 59) years. Nearly half (52.34%) were alcoholics and only 28.29% worked physical exercise. Of the total study subjects, 87.5% developed anxiety. Most patients with cancer were found in the first stage (58.38%) and gynecological-related cancer was the commonest type (17.97%) (Table 1).

**Table 1:** Background characteristics of study subjects in a study of prevalence and associated factors of adherence to guidelines in cancer pain management among adult patients evaluated at oncology unit, in among patients in the UoGCSH, Northwest Ethiopia 2021.

**Variables**	**Categories**	**Frequency (*n*)**	**Percentage (%)**
**Sociodemographic factors**			
Age in years	18–49	196	51.04
	≥50	188	48.96
Sex	Male	187	48.70
	Female	197	51.30
Marital status	Currently married	191	49.74
	Currently not married	193	50.26
Income status	Low	112	29.17
	Middle	248	64.58
	Higher	24	6.25
Residence	Urban	136	35.6
	Rural	246	64.4
**Psychosocial and behavioral factors**			
Alcohol drink	Not drink alcohol	183	47.66
	Drink alcohol	201	52.34
Cigarette smoking	Non-smoker	322	83.85
	Smoker	62	16.15
Chat chewing	Not used chat	281	73.18
	Used chat	103	26.82
Physical exercise	Vigorous	50	13.02
	Moderate	132	34.38
	Poor	202	52.6
Anxiety	No	48	12.5
	Yes	336	87.5
Social support	Poor	139	36.2
	Moderate	188	48.96
	Strong	57	14.84
**Clinical variables**			
Types of CA	Lung	16	4.17
	Breast	55	14.32
	Gynecological	69	17.97
	Hematologic	64	16.67
	GI	68	17.71
	Skin	15	3.91
	GU	25	6.51
	Endocrine	36	9.38
	Other	36	9.38
Stage	Stage I	223	58.38
	Stage II	70	18.32
	Stage III	52	13.61
	Stage IV	37	9.69
Metastasis	No	264	68.75
	Yes	120	31.25
Duration of diagnosis	≤ 4 months	233	60.68
	>4 months	151	39.32
Treatment modality	Chemotherapy	80	25.24
	Surgery	123	38.8
	Combination	114	35.96
Comorbidities	Absent	245	63.80
	Present	139	36.20
Analgesics graded	No analgesics	153	39.84
	None opioid	190	49.48
	Weak opioids	40	10.42
	strong opioids	1	0.26
Pain graded	No pain	205	53.39
	Mild pain	134	34.9
	Moderate pain	38	9.9
	Severe pain	7	1.82

### Prevalence and factors associated with poor adherence to guidelines in cancer pain management

The prevalence of poor adherence to guidelines in cancer pain management among patients in the University of Gondar Comprehensive Specialized Hospital, Northwest Ethiopia was 21.35% (95%CI: 17.53, 25.76). It was more prevalent among non-married patients (72.02%), patients with stage III (53.85%) and stage IV (62.16%) cancer, and those who have metastasis (84.11%) (Table 2).

**Table 2:** Bivariable and multivariable analyses of factors associated with poor adherence to guidelines in cancer pain management among patients in the UoGCSH, Northwest Ethiopia 2021.

**Variables**	**Categories**	**Poor adherence to guidelines**		**COR [95% CI]**	**AOR [95% CI]**
		**in cancer pain management**			
		**Yes (%)**	**No (%)**		
		***n* = 82 (21.35)**	***n* = 302 (78.65)**		
**Sociodemographic and behavioral factors**					
Age in years	18–49	36 (18.37)	160 (81.63)	1.00	1.00
	≥50	46 (24.47)	142 (75.53)	1.44 [0.88, 2.35]	0.91 [0.46, 1.78**]**
Marital status	Married	28 (14.66)	163 (85.34)	1.00	1.00
	Not married	54 (27.980)	139 (72.02)	2.26 [1.36, 3.76]	2.2 [1.15, 4.19]^*^
Cigarette smoking	Non-smoker	62 (19.25)	260 (80.75)	1.00	1.00
	Smoker	20 (32.26)	42(67.74)	2.14 [1.17, 3.91]	0.96 [0.38, 2.44]
Anxiety	No	15 (31.25)	33 (68.75)	1.00	1.00
	Yes	67(19.94)	269 (80.06)	0.55 [0.28, 1.06]	0.83 [0.29, 2.37]
Social support	Poor	36(25.9)	103 (74.1)	1.00	1.00
	Moderate	36(19.15)	152 (80.85)	0.68 [0.40, 1.15]	0.91 [0.42, 1.96]
	Strong	10 (17.54)	47 (82.46)	0.61 [0.28, 1.33]	0.54 [0.18, 1.62]
**Medical-related conditions**					
Types of cancer	Lung	8(50)	8 (50)	4.14 [1.15, 14.92]	0.40 [0.04, 3.48]
	Breast	9 (16.36)	46 (83.64)	0.81 [0.27, 2.41]	0.38 [0.04, 3.47]
	Gynecological	11 (15.94)	58 (84.06)	0.78 [0.27, 2.23]	0.45 [0.07, 2.76]
	Hematologic	10 (16.63)	54 (83.38)	0.77 [0.26, 2.22]	0.76 [0.09, 5.94]
	GI	17 (25)	51 (75)	1.38 [0.51, 3.72]	0.82 [0.06, 10.67]
	Skin	3 (20)	12(80)	1.03 [0.23, 4.69]	1.29 [0.15, 11.19]
	GU	5 (20)	20 (80)	2.07 [0.71, 6.09]	0.72 [0.06, 8.12]
	Endocrine	7 (19.44)	29 (80.56)	1.04 [0.28, 3.73]	0.29 [0.03, 2.51]
	Other	12 (33.33)	24 (66.66)	1.00	1.00
Stage of cancer	Stage I	32 (14.35)	191 (85.65)	1.00	1.00
	Stage II	11(15.71)	59 (84.29)	1.11 [0.53, 2.34]	1.01 [0.48, 2.14]
	Stage III	24 (46.15)	28(53.85)	5.12 [2.64, 9.91]	3.21 [1.64, 7.93]
	Stage IV	14 (37.84)	23 (62.16)	3.63 [1.69, 7.78]	1.63 [1.09, 5.81]
Metastasis	No	58 (24.89)	175 (75.11)	1.00	1.00
	Yes	24 (15.89)	127 (84.11)	4.51 [2.71, 7.53]	3.76 [1.83, 7.72]^**^
Duration of having CA	≤ 4 months	34(12.88)	230 (87.12)	1.00	1.00
	>4 months	48 (40)	72 (60)	0.57 [0.34, 0.96]	0.53 [0.26, 0.96]^*^
Treatment modality	Chemotherapy	19 (23.75)	61 (76.25)	1.00	1.00
	Surgery	12(9.76)	111 (90.24)	1.31 [0.68, 2.51]	0.91 [0.24, 3.51]
	Combination	33 (28.95)	81 (71.05)	0.35 [0.16, 0.76]	0.43 [0.11, 1.76]
Comorbidities	Absent	46 (18.78)	199 (81.22)	1.00	1.00
	Present	36 (25.9)	103 (74.1)	1.51 [0.92, 2.48]	1.05 [0.54, 2.07]

AOR, adjusted odds ratio; COR, Crude odds ratio; CI, confidence interval.

* = P < 0.05,

** = P < 0.01, ^***^ = P < 0.001.

**Figure 1:**
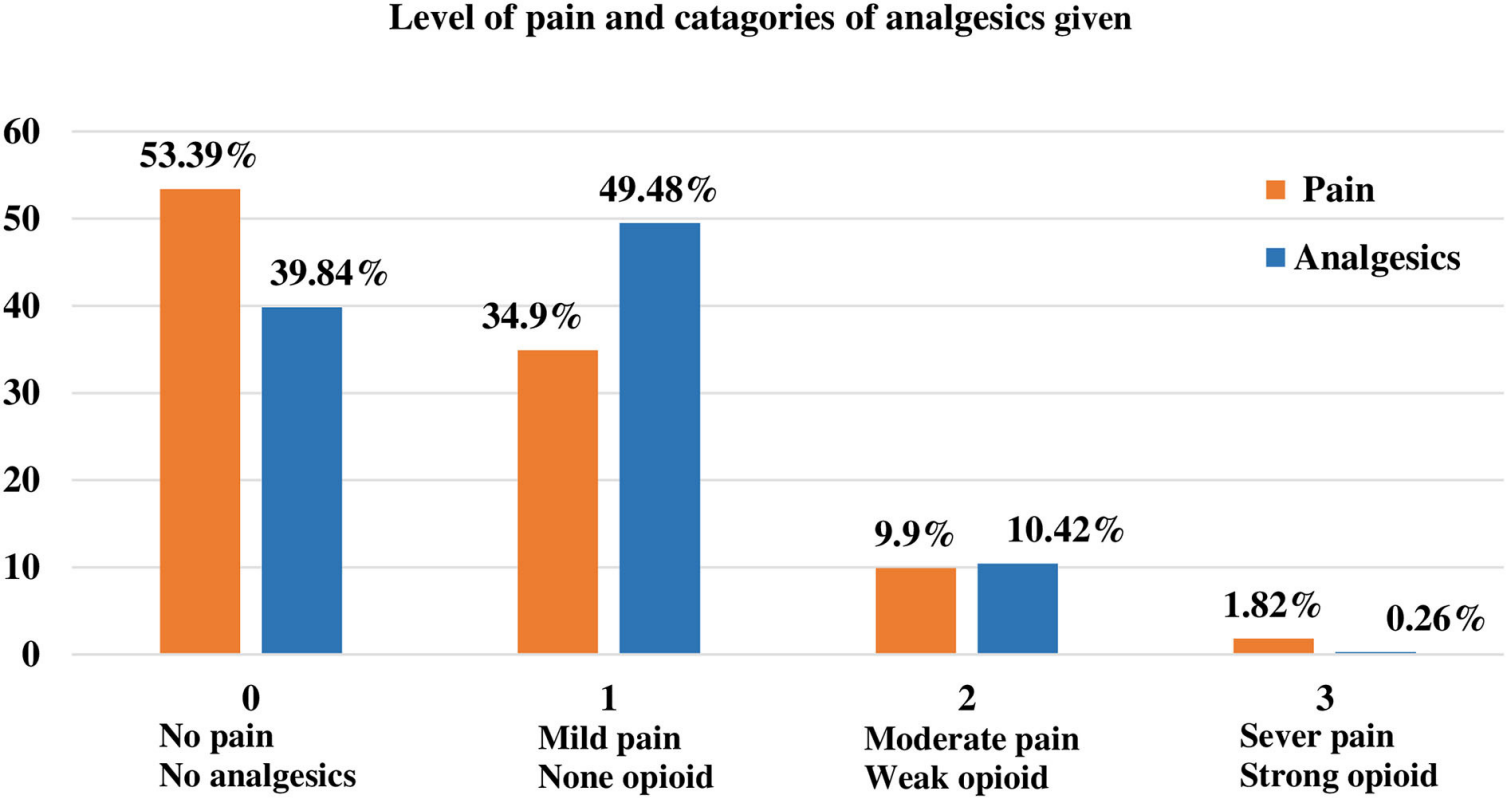
The proportion of the level of pain and categories of analgesics were given.

**Figure 2:**
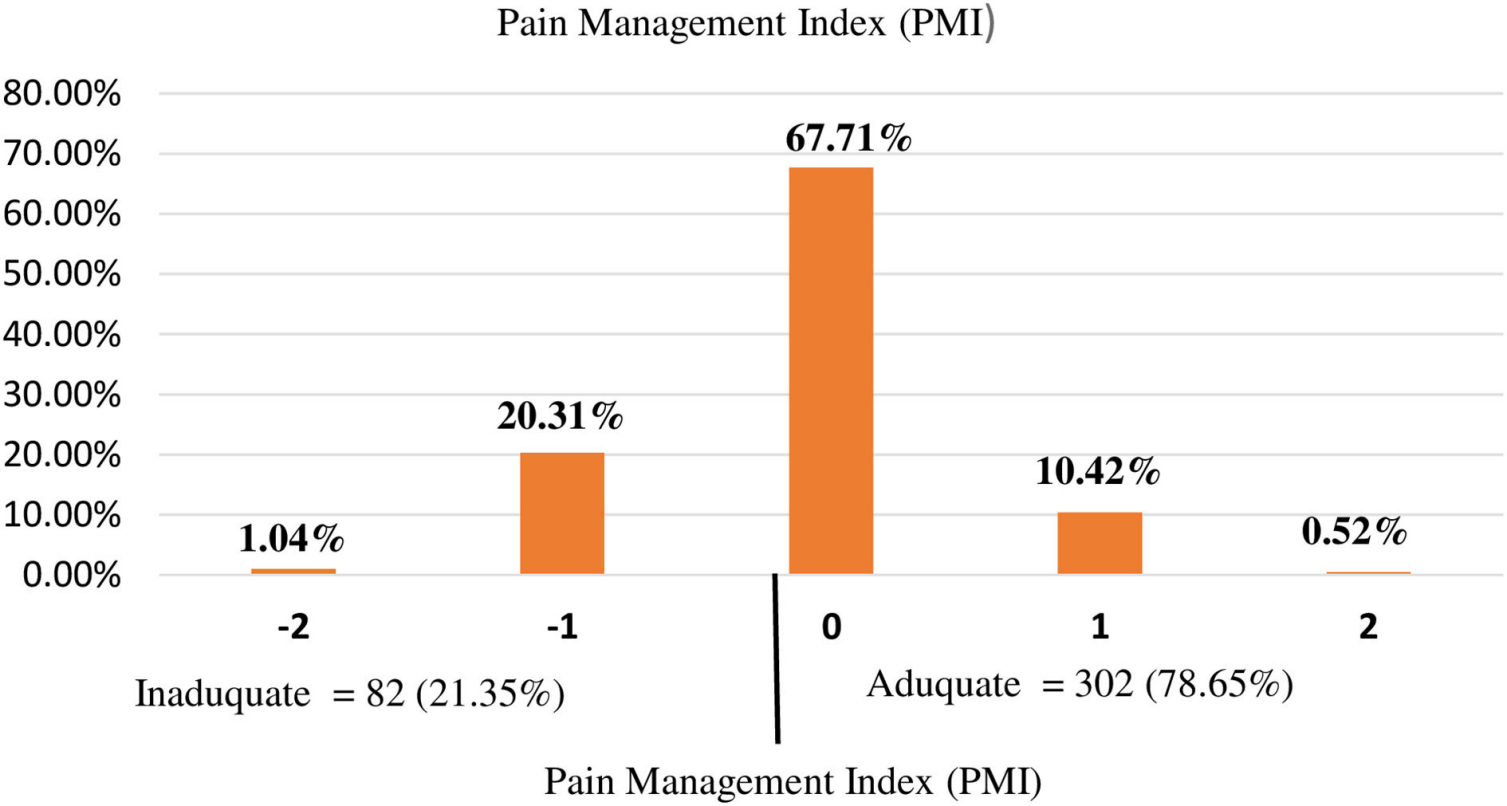
The proportion of pain management index (PMI).

In our study, the disproportional analgesics given for the appropriate level of pain were relatively high in grade 0 and grade 1 patients. As shown in Figure 1, of the total study subjects, 55.39% of patients had no pain but only 39.84% of patients were not taken analgesics. This means that an extra 15.55% of patients were taken analgesics without feeling pain (Figures 1, 2).

All variables were analyzed using univariate analysis to assess the association between each variable and poor adherence to guidelines in cancer pain management using univariate logistics regression analysis. Then, variables that have a *p* ≤ 0.2 in the univariate analysis were taken to multivariate analysis. But variables such as sex, income status, residence, alcohol intake, chat-related substance intake, and doing physical exercise had a *p* > 0.2 in univariate analyses and exclude from multivariable analyses. On the other hand, out of those variables treated under multivariate analysis, marital status, stage of cancer, presence of metastasis, and duration of knowing having cancer had statistically significantly associated with poor adherence to guidelines in cancer pain management.

Patients who were not married were a two times higher chance of having poor adherence to guidelines in cancer pain management as compared to those who were married [AOR = 2.2; 95%CI: 1.15, 4.19]. The odds of having poor adherence to guidelines in cancer pain management among stage III [AOR = 3.21; 95%CI: 1.64, 7.93] and stage IV [AOR = 1.63; 95%CI: 1.09, 5.81] patients were 3.2 and 1.6 times higher than stage I patients, respectively.

Patients who have metastasis cancer were 3.8 times higher having poor adherence to guidelines in cancer pain management as compared to those who did not have metastasis [AOR = 3.76; 95%CI: 1.83, 7.72]. On the other hand, patients who know their diagnosis 4 months ago before starting treatment were 47% less likely to have poor adherence to guidelines in cancer pain management as compared to those within a 4-month diagnosis [AOR = 0.53; 95%CI: 0.26, 0.96] (Table 2).

## Discussion

Cancer pain is one of the most important deleterious and distressing symptoms suffered by patients with cancer besides the other symptoms which disturb their quality of life (8, 19). Trying to proceed with inadequately managed cancer pain can cause nerve changes that could make the pain harder to control in the future (28). People with chronic cancer pain might have times when their medicines do not control the pain all the time which is called breakthrough pain (28). Moreover, poor cancer pain management harms the physical, psychological, spiritual, and emotional wellbeing of patients with cancer (29, 30). For this reason, alleviating pain is a primary goal of prognosis of cancer pain management, and pain symptoms must be prevented, treated as a priority, and considered an independent part of cancer management (9).

This study aimed to assess the prevalence of poor adherence to guidelines in cancer pain management and possible associated factors among adult patients with cancer in UoGCSH, Northwest Ethiopia. Based on this, the prevalence of poor adherence to guidelines in cancer pain management among adult patients with cancer in the UoGCSH was 21.35% (95%CI: 17.53, 25.76). Factors such as marital status, stage of cancer, presence of metastasis, and duration of knowing having cancer had statistically significantly associated with having poor adherence to guidelines in cancer pain management.

The prevalence of poor adherence to guidelines in cancer pain management in our study (21.35%) is in line with a study in Italy, 25.3% of patients were under treatment (19). But this study is higher than a study in Libya, 3.87% of patients were associated with poor adherence to guidelines in cancer pain management (8). On the other hand, this study is lower than a study conducted in a similar setting (UoGCSH) 5 years before (65%) (16) and Ayder Comprehensive Specialized Hospital (43.95%) (18) in Ethiopia, in Japan (70%) (7), and in Punjab, India (77%) (9). This might be due to the difference in socioeconomic status, the patient's inability to communicate the intensity of his or her pain, and the patient's culture (9). Culture can significantly influence patients with cancer coping behaviors, pain experience, and adherence to a recommended pain management plan (31). Therefore, providing culturally appropriate care is an essential element of effective cancer pain management for patients from culturally and linguistically diverse backgrounds like Ethiopia. Poor guidelines dissemination and lack of homogeneous service development for patients with cancer pain have also contributed (19). Studies showed that patients with lower socioeconomic status had the highest risk of under treatment (19). In many developing countries, morphine and other analgesics are not available or are not in regular supply, but in developed countries, have easy access to healthcare and required prescription drugs, and culture exists in which taking pain medications is not perceived negatively (9).

In our study, patients who were not married have a higher chance of having poor adherence to guidelines in cancer pain management as compared to those married. This is in line with a study in Ayder Comprehensive Specialized Hospital, Ethiopia (18). Research showed that being married greatly increases patients' chances of survival from cancer and better pain treatment since they were more likely to listen to their doctor's advice and adhere to their medication schedules (32). On the other hand, married patients and patients having a partner were more likely to have low loneliness (33), as a result, they experience less pain than unmarried patients (34).

In this study, as the stage of cancer increases to stage III and stage IV, the odds of having good adherence to treatments for cancer pain become higher. This is in line with a study in Ayder Comprehensive Specialized Hospital, Ethiopia (18). But a study in Japan showed that patients with non-advanced cancer were more likely to receive inadequate treatment than those with advanced cancer (7). As the stage of cancer increases, the disease itself or the treatment given can result in nerve damage (28). At this time a liberal use of opioids for patients is always suggested, but, a conservative approach to opioid use with advanced-stage cancer may result in the inadequate treatment of cancer pain (9). Moreover, adjuvant and non-pharmacological treatments are more recommended for the therapy for treating patients with advanced-stage cancer with persistent pain in combination with analgesics or other medications (11).

In this study, patients who have metastasis cancer have a higher risk to have poor adherence to guidelines in cancer pain management as compared to those who did not have metastasis. This is supported by a study in Ayder Comprehensive Specialized Hospital, Ethiopia (18), and a study at Gondar University Hospital, Ethiopia (16). This is because of that, the most common type of pain is related to metastases, especially when metastasis to bone occurs, and as a result, might develop pain breakthrough (35).

On the other hand, in this study patients who knows their diagnosis 4 months ago were less likely to have poor adherence to guidelines in cancer pain management as compared to patients who know their diagnosis within 4 months. This is in line with a study in Italy (19). This might be that patients adapt to cancer symptoms and medications as the time of knowing having cancer increases.

The strengths of this study come from the use of a relatively large number of samples as compared to the previous study conducted in the same area or different settings, which makes it representative of populations of study settings. Therefore, it can be generalized to all patients in the UoGCSH during the study period. Although the usefulness of the PMI is proved by a large number of studies, some drawbacks are well-known. It takes into account only one characteristic of pain (the intensity) and the grade of ant pain used but does not reflect other pain characteristics, opioid titration, route of administration, adjuvant therapies, or the use of non-pharmacological therapies.

## Conclusion

The prevalence of poor adherence to guidelines in cancer pain management among adult patients with cancer in UoGCSH Northwest Ethiopia is relatively low as compared with studies in Ethiopia Ayder Hospital and UoGCSH (5 years before), in Japan, and in Punjab, India.

Factors such as patients who were not married and who have metastasis, and being patients with stage III and stage IV cancer had a significant positive association to have poor adherence to guidelines in cancer pain management, on the other hand, patients who know their diagnosis 4 months ago have good adherence to guidelines in cancer pain management.

Patients with high stage and metastasis need care from pain specialists early on in the diagnosis of pain. The hospital should reassure the diagnosis of cancer for the patient before they started the treatment.

## Data availability statement

The raw data supporting the conclusions of this article will be made available by the authors, without undue reservation.

## Ethics statement

The studies involving human participants were reviewed and approved by University of Gondar IRB. Written informed consent to participate in this study was provided by the participants' legal guardian/next of kin.

## Author contributions

AK, DB, HW, MM, and HA: conceptualization, formal analysis, methodology, supervision, and writing original draft. AK, DB, ET, EM, and YW: data curator, investigation, and validation. AK, DG, ET, EM, YW, HW, MM, and HA: resources. AK, DB, HW, MM, HA, ET, EM, and YW: software. AK, HW, MM, HA, and DB: visualization. All authors contributed to the article and approved the submitted version.

## Conflict of interest

The authors declare that the research was conducted in the absence of any commercial or financial relationships that could be construed as a potential conflict of interest.

## Publisher's note

All claims expressed in this article are solely those of the authors and do not necessarily represent those of their affiliated organizations, or those of the publisher, the editors and the reviewers. Any product that may be evaluated in this article, or claim that may be made by its manufacturer, is not guaranteed or endorsed by the publisher.

## Abbreviations

AOR: adjusted odds ratio
COR: crude odds ratio
CA: cancer
CR: cancer-related pain
PMI: pain management index
OPD: outpatient department
UOGCSH: University of Gondar Comprehensive Specialized Hospital.
